# Time-to-diagnosis and symptoms of myeloma, lymphomas and leukaemias: a report from the Haematological Malignancy Research Network

**DOI:** 10.1186/2052-1839-13-9

**Published:** 2013-10-31

**Authors:** Debra A Howell, Alexandra G Smith, Andrew Jack, Russell Patmore, Una Macleod, Emma Mironska, Eve Roman

**Affiliations:** 1 Epidemiology and Cancer Statistics Group, Department of Health Sciences, University of York, York YO10 5DD, UK; 2 Haematological Malignancy Diagnostic Service, Bexley Wing, St James University Hospital, Leeds LS9 7TF, UK; 3 Queens Centre for Oncology, Castle Hill Hospital, Cottingham, Hull HU16 5JQ, UK; 4 Hull York Medical School, University of Hull, Hertford Building, Hull HU6 7RX, UK; 5 St James University Hospital, Leeds LS9 7TF, UK

**Keywords:** Haematological malignancies, Help-seeking, Diagnostic delay

## Abstract

**Background:**

Prior to diagnosis, patients with haematological cancers often have multiple primary care consultations, resulting in diagnostic delay. They are less likely to be referred urgently to hospital and often present as emergencies. We examined patient perspectives of time to help-seeking and diagnosis, as well as associated symptoms and experiences.

**Methods:**

The UK’s Haematological Malignancy Research Network (http://www.hmrn.org) routinely collects data on all patients newly diagnosed with myeloma, lymphoma and leukaemia (>2000 annually; population 3.6 million). With clinical agreement, patients are also invited to participate in an on-going survey about the circumstances leading to their diagnosis (presence/absence of symptoms; type of symptom(s) and date(s) of onset; date medical advice first sought (help-seeking); summary of important experiences in the time before diagnosis). From 2004–2011, 8858 patients were approached and 5038 agreed they could be contacted for research purposes; 3329 requested and returned a completed questionnaire. The duration of the total interval (symptom onset to diagnosis), patient interval (symptom onset to help-seeking) and diagnostic interval (help-seeking to diagnosis) was examined by patient characteristics and diagnosis. Type and frequency of symptoms were examined collectively, by diagnosis and compared to UK Referral Guidelines.

**Results:**

Around one-third of patients were asymptomatic at diagnosis. In those with symptoms, the median patient interval tended to be shorter than the diagnostic interval across most diseases. Intervals varied markedly by diagnosis: acute myeloid leukaemia being 41 days (Interquartile range (IQR) 17–85), diffuse large B-cell lymphoma 98 days (IQR 53–192) and myeloma 163 days (IQR 84–306). Many symptoms corresponded to those cited in UK Referral Guidelines, but some were rarely reported (e.g. pain on drinking alcohol). By contrast others, absent from the guidance, were more frequent (e.g. stomach and bowel problems). Symptoms such as tiredness and pain were common across all diseases, although some specificity was evident by sub-type, such as lymphadenopathy in lymphoma and bleeding and bruising in acute leukaemia.

**Conclusions:**

Pathways to diagnosis are varied and can be unacceptably prolonged, particularly for myeloma and some lymphomas. More evidence is needed, along with interventions to reduce time-to-diagnosis, such as public education campaigns and GP decision-making aids, as well as refinement of existing Referral Guidelines.

## Background

In comparison to many other cancers, the pathway to diagnosis of haematological malignancies (leukaemias, lymphomas and myeloma) can be fraught with difficulty, and is often associated with excessive time between symptom onset, help-seeking and diagnosis; multiple primary care consultations before referral to secondary care; and an increased chance of being diagnosed after emergency admission [1-17]. Diagnostic delay is considered to increase complications in patients with some haematological malignancies [8], and a recent review specifically identified these as diseases for which early diagnosis could improve outcome [18]. In Great Britain a recent study estimated that around 3500 deaths occurring within five years of diagnosis of a haematological malignancy could be avoided if survival matched that of the rest of Europe [19].

Haematological malignancies comprise a heterogeneous group of over 60 cancer sub-types, many of which have unique clinical pathways and outcomes [20]. As a group these cancers are relatively common, accounting for around one in ten of all new cancer diagnoses in the developed world [21,22]. However, unlike some cancers, their clinical presentation is recognised as being broad and ill-defined, particularly in terms of initial symptoms; which may be non-specific, difficult to differentiate from those of benign, self-limiting conditions, and associated with a long prodrome [2,7]. Early diagnosis of haematological malignancies therefore poses exacting challenges for patients and clinicians; patients must determine when to seek help and, when they do, practitioners must identify the symptoms of potential malignancy and make appropriate and timely referrals to secondary care.

Ensuring early diagnosis of all cancers, including haematological malignancies, has been a key priority for the UK Department of Health for over a decade [23-27]. Initiatives such as the production of Referral Guidelines for Suspected Cancer to help general practitioners (GPs) identify cancer symptoms early, and waiting-time targets to ensure rapid diagnosis and treatment, are now firmly embedded in primary and secondary healthcare systems [28]. In terms of haematological malignancies, a single list of symptoms has been developed to guide GPs in their identification of patients with constellations of these, which may indicate potential disease. Existing evidence about symptoms of haematological malignancies, however, has been largely derived from expert committee reports [28] or from studies that have focused on pre-determined symptoms or clinical parameters (blood and other diagnostic test results) [9,10,29-31]; although several often smaller studies do exist that have examined complete symptom profiles [7,8,11,12,32-35]. The aim of this study was to examine time to help-seeking and diagnosis of haematological malignancies, as well as associated symptoms, from the patient perspective.

## Methods

Covering a population of 3.6 million that is broadly representative of the UK as a whole, this study was conducted within the robust infrastructure of the Haematological Malignancy Research Network’s (http://www.hmrn.org) on-going patient cohort [36,37]. Established in 2004, HMRN is a collaboration between the clinical haematology network, researchers at the University of York and the Haematological Malignancy Diagnostic Service (http://www.hmds.info), which diagnoses all haematological malignancies in the area coding to the latest WHO classification scheme [20]. More than 2000 patients are registered annually and demographic, prognostic and treatment data are routinely abstracted from their medical records. HMRN has full ethical approval and Section 251 exemption to collect data for audit and research purposes.

In addition, and with permission from their clinical teams, patients are asked to consent to being approached in the future for further research purposes. Reflecting the diversity of disease sub-types, different consenting strategies are used (face-to-face for in-patients, postal for out-patients etc.). At this time, patients (≥ 18 years) are also given an information leaflet inviting them to take part in our on-going survey about symptoms and help-seeking. Those that agree are sent a questionnaire asking: *'Did you have any symptoms before you were diagnosed with your****present****illness?’* to which the response is documented by ticking 'Yes’ or 'No’. People that tick 'Yes’ are asked to list all their symptom(s) during this time and the exact (or if not approximate) date of onset of each. Finally, patients are asked to provide the date that medical advice was first sought (help-seeking) for any of the symptoms that they have listed. To avoid leading questions patients’ report in free-text, using their own words and phrases, rather than ticking pre-selected symptoms. We do not ask that symptoms reported are limited to those that the patients are sure relate only to their haematological malignancy; this is because of the difficulties inherent in such decisions. Importantly, the questionnaire also contains a text box in which patients can tell us anything else they consider important in relation to their disease pathway.

This report summarises information on time to help-seeking and diagnosis, as well as symptoms, collected over seven years, 2004–11. Three time intervals were examined in symptomatic patients; the total interval (from date of first symptom onset to diagnosis); the patient interval (from date of first symptom onset to first help-seeking); and the diagnostic interval (from date of first help-seeking to diagnosis and including the time when the patient’s care is being managed in primary and/or secondary care) [38]. These data are examined by patient characteristics and diagnosis and are presented as medians and interquartile ranges (IQRs). Symptoms are examined in total and by diagnostic group and findings compared to those cited in the UK Referral Guidelines [28]. All analyses were conducted using Stata version 12 [39] and standard descriptive methods were applied. Finally, information reported in the free text box of the questionnaire by individuals about their diagnostic experiences was studied in detail; examples were then drawn together for illustrative purposes, in order to highlight the variations and difficulties inherent in this process.

## Results

During the seven year period September 2004 to August 2011, 5038 (57%) of the 8858 patients approached agreed to be contacted again, and 3329 (66% of the 5038, 38% of the 8858) requested and returned a completed questionnaire (Table 1). No marked demographic or diagnostic differences were observed between subjects who agreed to complete a questionnaire and those who did not.

**Table 1:** Characteristics of patients diagnosed with a haematological malignancy: Haematological Malignancy Research Network (HMRN), 2004–2011

	**Contacted N (%)**	**Consented to further contact N (%)**	**Questionnaire returned N (%)**	**Symptoms N (%)**		**Interval - Median Days (25–75 percentile – interquartile range)**		
				**No**	**Yes**	**Total**^**2**^	**Patient**^**3**^	**Diagnostic**^**4**^
Total	8858 (100)	5038 (100)	3329 (100)	993(29.8)	2336 (70.2)	123 (55–277)	17 (1–86.5)	65 (26–155)
**Sex:**								
Males	4938 (55.7)	2840 (56.4)	1865 (56)	617 (33.1)	1248 (66.9)	108 (52–252)	16 (1–75)	62 (24–152)
Females	3920 (44.3)	2198 (43.6)	1464 (44)	376 (25.7)	1088 (74.3)	138 (62–315)	26 (1–91)	72 (29–158)
**Age at diagnosis (years):**								
Median age (Range)	69.2 (18.1-99.7)	67.7 (18.1-96.8)	67.4 (18.1-95.2)	66.3 (18.1-95.2)	69.9 (19.2-94.7)	-	-	-
< 40	664 (7.5)	330 (6.6)	216 (6.5)	25 (11.6)	191 (88.4)	120.5 (47–251)	23.5 (1–92)	55.5 (20–137)
40-59	1821 (20.6)	1138 (22.6)	773 (23.2)	178 (23.0)	595 (77.0)	124 (59–258)	30 (1–85)	64 (27–144)
60-69	2087 (23.6)	1362 (27)	921 (27.7)	295 (32.0)	626 (68.0)	119.5 (56–269)	17 (1–91)	67 (26–167)
70-79	2701 (30.5)	1506 (29.9)	1018 (30.6)	350 (34.4)	668 (65.6)	122 (57–311.5)	16 (1–74)	66 (27–142.5)
80+	1585 (17.9)	702 (13.9)	401 (12)	145 (36.2)	256 (63.8)	139 (48–354)	16 (1–78)	73 (28–189)
**Diagnostic group**^**1**^**:**								
Diffuse large B-cell lymphoma	1342 (15.2)	771 (15.3)	503 (15.1)	52 (10.3)	451 (89.7)	98 (53–192)	9 (1–42)	69 (37–134)
Myeloma	1263 (14.3)	755 (15)	493 (14.8)	152 (30.8)	341 (69.2)	163 (84–306)	31 (1–122)	83 (34–167)
Follicular lymphoma	660 (7.5)	413 (8.2)	284 (8.5)	51 (18.0)	233 (82.0)	112 (61–250)	17 (1–76)	65 (36–125.5)
Myeloproliferative neoplasms	1183 (13.4)	674 (13.4)	430 (12.9)	205 (47.7)	225 (52.3)	215 (84–539)	31 (1–168)	77 (27–239)
Marginal zone lymphoma	616 (7)	384 (7.6)	247 (7.4)	68 (27.5)	179 (72.5)	172 (77–385)	30 (1–153)	86.5 (27–193.5)
Myelodysplastic syndromes	720 (8.1)	424 (8.4)	267 (8)	97 (36.3)	170 (63.7)	147 (55–393)	16 (1–89)	88 (24–249)
Hodgkin lymphoma	574 (6.5)	292 (5.8)	193 (5.8)	26 (13.5)	167 (86.5)	158 (84–288)	30 (2–77)	87 (40–166)
Chronic lymphocytic leukaemia	650 (7.3)	344 (6.8)	265 (8)	140 (52.8)	125 (47.2)	86 (38–238)	22 (1–85)	42 (12–88)
Acute myeloid leukaemia	538 (6.1)	249 (4.9)	161 (4.8)	37 (23.0)	124 (77.0)	41 (17–85)	13 (1–47)	10 (5–32)
Chronic myeloid leukaemia	207 (2.3)	143 (2.8)	95 (2.9)	28 (29.5)	67 (70.5)	96 (32–169)	33.5 (4.5-127.5)	9 (5–52)
Mantle cell lymphoma	159 (1.8)	96 (1.9)	65 (2)	13 (20.0)	52 (80.0)	104 (58–258)	21.5 (1–69.5)	61 (29–144)
Lymphoproliferative disorder NOS	322 (3.6)	153 (3)	97 (2.9)	53 (54.6)	44 (45.4)	324 (48–578)	16 (1–30)	185 (27–417)
T-cell lymphoma	148 (1.7)	72 (1.4)	52 (1.6)	10 (19.2)	42 (80.8)	175 (70–306)	18.5 (1–92)	71 (39–167)
Acute lymphoblastic leukaemia	96 (1.1)	50 (1)	37 (1.1)	8 (21.6)	29 (78.4)	32.5 (17–64)	16 (2–26)	12.5 (3–32)
Myelofibrosis	91 (1)	54 (1.1)	35 (1.1)	12 (34.3)	23 (65.7)	314 (76–742)	5 (1–66)	93 (25–320)
Myelodysplastic/Myeloproliferative neoplasms	125 (1.4)	66 (1.3)	41 (1.2)	18 (43.9)	23 (56.1)	421 (139–709)	31 (16–308)	128 (27–840)
T-cell leukaemia	62 (0.7)	37 (0.7)	32 (1)	13 (40.6)	19 (59.4)	502 (75–761)	30 (3–153)	61 (37–367)
Hairy cell leukaemia	65 (0.7)	43 (0.9)	26 (0.8)	10 (38.5)	16 (61.5)	88.5 (44–194)	35 (1–138)	16 (10–32)
Burkitt lymphoma	37 (0.4)	18 (0.4)	6 (0.2)	0 (0.0)	6 (100.0)	67.5 (34–136)	19 (4.5-30.5)	36 (11–82.5)

^1^Ordered by the absolute numbers of patients reporting symptoms; ^2^Total interval – symptom onset to diagnosis; ^3^Patient interval – symptom onset to help-seeking; ^4^Diagnostic interval – help-seeking to diagnosis.

Just over two-thirds (2336) of the 3329 patients who returned a completed questionnaire reported that they had one or more symptoms before diagnosis. The diagnostic categories listed in Table 1 are ordered according to the absolute numbers of patients reporting symptoms; ranging from 451 with diffuse large B-cell lymphoma through to six with Burkitt lymphoma. The likelihood of experiencing symptoms varied markedly by disease sub-type, as can be seen from Table 1, with around half of patients with some of the more slowly progressing conditions, such as chronic lymphocytic leukaemia and myeloproliferative neoplasms, being asymptomatic at diagnosis (52.8% and 47.7% respectively). In contrast, nearly all patients with more aggressive diseases, such as diffuse large B-cell and Hodgkin lymphoma reported symptoms (89.7% and 86.5% respectively). Indeed, many asymptomatic patients with chronic lymphocytic leukaemia reported being diagnosed incidentally via blood tests at routine health checks, or check-ups for other comorbidities, rather than presenting with symptomatic disease.

In symptomatic patients, differences in time to help-seeking and diagnosis were observed by sex and age. Among those with symptoms, women had, on average, longer intervals than men, and those aged ≥80 years tended to have longer total intervals (symptom onset to diagnosis) than younger patients and this was largely driven by the length of the diagnostic interval (help-seeking to diagnosis). As with the occurrence of symptoms, there is marked variation in the duration of intervals by diagnosis (Table 1). As might be expected, the total interval was shortest for acute myeloid leukaemia at 41 days (interquartile range (IQR) 17–85). The lymphomas tended to have longer intervals with the more aggressive diffuse large B-cell lymphoma being diagnosed soonest at 98 days (IQR 53–192) and marginal zone (generally the most indolent) having the greatest delay at 172 days (IQR 77–385). The total interval for myeloma was also prolonged at 163 days (IQR 84–306) and myeloproliferative neoplasms, again being particularly indolent, were found to have the longest overall time-to-diagnosis at 215 days (IQR 84–539). For the vast majority of conditions the average patient interval (symptom onset to help-seeking) was considerably shorter than the diagnostic interval. The main exceptions to this were chronic myeloid leukaemia, where the patient and diagnostic intervals were 33.5 days (IQR 4.5-127.5) and 9 days (IQR 5–52) respectively and acute myeloid leukaemia, being 13 days (IQR 1–47) and 10 days (IQR 5–32) respectively. In contrast, for diffuse large B-cell lymphoma, the most common of the lymphomas, the diagnostic interval accounted for an extremely large proportion of the time-to-diagnosis, having a patient interval of 9 days (IQR 1–42) and diagnostic interval of 69 days (IQR 37–134).

**Figure 1:**
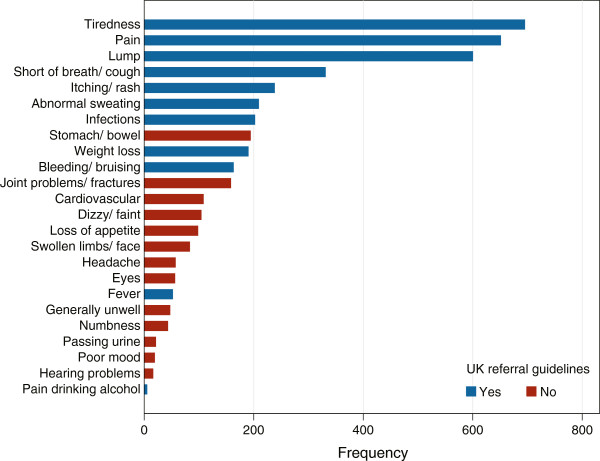
**Distribution of symptoms.** All symptoms are coded once only. *Pain*: includes musculoskeletal, abdominal, chest and other; *Infections*: include throat, chest, common cold, flu (like symptoms), mouth sores, skin infections and other; *Stomach/bowel*: nausea, vomiting, bloated, indigestion, diarrhoea and other; *Bruising/bleeding*: includes nosebleeds, bleeding from bowel/stomach, gums/mouth and other; *Cardiovascular*: includes abnormal blood pressure, abnormal heart beat, stroke, deep vein thrombosis and other.

Information on symptom frequency across all haematological malignancies combined is presented in Figure 1. In line with UK Referral Guidelines (blue bars), symptoms most frequently reported were tiredness, pain, lump, shortness of breath/cough, skin problems, abnormal sweating and infections. However, certain listed symptoms were mentioned comparatively infrequently, most notably pain when drinking alcohol, which was only reported by five patients – all with lymphoma. By contrast, as can be seen from Figure 1 (red bars), patients identified a range of other symptoms, including for example, stomach/bowel problems, joint problems and fractures, cardiovascular problems, dizziness and loss of appetite. No differences were detected in time-to-diagnosis between those that reported having symptoms cited in the UK Referral Guidelines and those that did not (data not shown).

**Figure 2:**
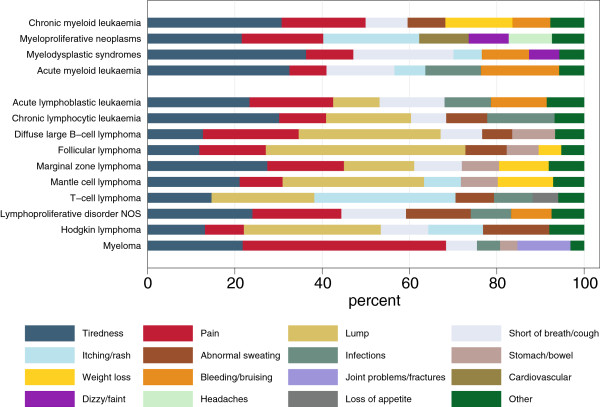
Distribution of symptoms by diagnostic group.

A number of the reported symptoms, such as tiredness and pain, occurred across all diseases (Figure 2). The type of pain reported varied, however, by diagnosis, with musculoskeletal pain being particularly pronounced in myeloma, abdominal pain being common in the non-Hodgkin lymphomas and chest pain in patients with acute leukaemia and myelodysplastic syndrome (data not shown). Furthermore, in contrast to traditional perceptions of painless lymphadenopathy, some patients reported otherwise, particularly for Hodgkin lymphoma. For other symptoms there was greater specificity by sub-type: bruising/bleeding and shortness of breath/cough in acute myeloid leukaemia and myelodysplastic syndromes; lymphadenopathy (usually reported as a lump) in lymphoma; joint problems and fractures in myeloma. However, with around half of all patients only reporting one symptom, there were no obvious constellations by diagnostic group (data not shown). We were unable to match any of the reported symptoms with any of those in the Referral Guidelines in 10% of patients, and this was most common in patients with myeloproliferative neoplasms. In addition to skin problems (which are included in the Referral Guidelines) patients with myeloproliferative neoplasms were most likely to report headaches, cardiovascular problems and feeling dizzy/faint (none of which are included in the Guidelines).

The challenges and variations associated with diagnosing haematological cancers, particularly myeloma and lymphoma, are clear from the List of self-reported experiences presented, which have been drawn together for illustrative purposes. These accounts range from patients acknowledging that they were diagnosed incidentally after a routine blood test, without experiencing any symptoms, to those reporting particularly poor experiences in terms of multiple symptoms, repeated consultations, emergency presentation and prolonged time-to-diagnosis. A wide variety of reasons for delayed help-seeking are highlighted in these excerpts including: musculoskeletal pain on a background of existing bone/joint problems; having an abnormal lump with no pain; and having symptoms which were intermittent. However, poorer experiences did not always correlate with longer time-to-diagnosis; and some patients were diagnosed quickly, but reported being acutely ill and repeatedly consulting GPs or presenting in emergency departments during this time.

### List of self-reported experiences

Diffuse large B-cell lymphoma, aged 30–40 years

**Symptoms:** Pain in left shoulder and left side of chest, not able to lie down, tiredness, breathlessness, cough, weight loss, coughing up blood.

**Free text:** “Went to the doctors at least 8 times. Kept telling me it would not be anything serious as I was too young. I demanded an x-ray in (month) as symptoms kept getting worse.”

**Interval:** Patient (i.e. symptom onset – help-seeking) - immediate; diagnostic (i.e. help-seeking – diagnosis) - 5 months.

Diffuse large B-cell lymphoma, aged 70–80 years

**Symptoms:** Tiredness, lump in neck.

**Free text:** “Initially, the lump appeared and disappeared gradually getting bigger and harder each time. There was no pain or discomfort associated with it at any time.”

**Interval**: Patient - 3 months; diagnostic - 2 months.

Myeloma, aged 50–60 years

**Symptoms:** Aches in bones/joints, general mood change.

**Free text:** “I think most of the time my doctor at doctors thought I was just a hypochondriac and putting it on as I suffer from long term post-traumatic stress disorder.”

**Interval:** Total (i.e. symptom onset – help-seeking) - 1 year.

Myeloma, aged 60–70 years

**Symptoms:** Backache, tiredness, poor skin and hair.

**Free text:** “I had two hip replacements in the past. I assumed that the backache in (month) was due to something wrong with these. I was prescribed (drug) and later (drug). My doctor sent me for a hip x-ray at the hospital only after the physio I was seeing asked (doctor) directly. This came back with no problem with the hips. As regards the backache, this just got worse and worse and still no referral to an ortho was arranged until I had reached rock bottom physically and mentally. The ortho blood tests revealed high calcium levels and I was admitted as an emergency (month).”

**Interval:** Patient - 1 month; diagnostic - 1 year.

Myeloproliferative neoplasm, aged 30–40 years

**Symptoms:** Itchiness (mainly legs, also arms, back and chest), red/brown speckles on toes and ankles, migraine.

**Free text:** “Although I was aware of the symptoms for some time I had put them down to other potential causes e.g. itchiness seemed to follow certain foods, speckles on toes/ankles looked like freckles, migraines came when tired following busy periods at work and more recently (date and event) when I also felt faint at work on a couple of occasions when particularly tired. The itchiness had become more regular and more severe in that I was unable to sleep on some nights (which could be several nights in a row) obviously adding to the tiredness. However, the symptoms were more of an inconvenience that a particular worry and I didn’t specifically seek medical advice. I just mentioned them when visiting the doctor for an unrelated knee pain. The subsequent blood test (in light of the speckles) uncovered the blood disorder.”

**Interval:** Patient - 18 months; diagnostic – 1 year.

Myeloproliferative neoplasm, aged 60–70

**Symptoms:** None reported.

**Free text:** “I didn’t seek medical help for my blood disorder – it was detected through the regular blood tests which I have as part of the monitoring of my diabetes.”

**Interval:** Incidental finding – no delay.

Acute myeloid leukaemia, aged 40–50 years

**Symptoms:** Lower back ache, unexplained bruises, feeling weak and tired.

**Free text:** “On (date), the day I first went to the Doctors, I was able to carry out my normal daily duties e.g. shopping, walking about, cleaning etc. The only reason I went to the Doctors was because of a few unexplained bruises. I had a period at the time which was a lot heavier than my normal ones. This got worse throughout the week, so I think I would have noticed something was wrong had I not already been to the doctors. I was admitted to hospital (day after help-seeking).”

**Interval:** Patient – 2 months; diagnostic – 1 day.

## Discussion

This paper presents results from a study examining self-reported time to help-seeking and diagnosis, as well as symptoms in a large cohort of patients with haematological cancers. Substantial variation was noted in the experiences reported by patients and this was largely driven by diagnostic sub-type. This is because sub-type generally determines disease aggressiveness, the manifestation of symptom(s) and the speed with which these exacerbate. Thus time-to-diagnosis was shortest for acute myeloid leukaemia, which is generally perceived as the most aggressive, acute and rapidly progressive disease, longer for the lymphomas and particularly prolonged for myeloma. The large number of patients without any symptoms (30% overall, rising to around half in particularly indolent conditions) often reported that they had been diagnosed incidentally when blood tests were taken to monitor other comorbidities or at routine health checks carried out at GP surgeries. In this respect, the diagnostic sub-types of those less likely to experience symptoms in our study correspond with the diseases that can be identified by blood testing alone (e.g. chronic lymphocytic leukaemia and myeloproliferative neoplasms).

A myriad of different symptoms were reported and although some, such as tiredness and pain, were common across diseases, there was some specificity by sub-type. Tiredness, perhaps occurring as a consequence of anaemia was expected, however, the frequency with which pain was reported was surprising and it is possible that pain may have been previously underestimated. Although many of the symptoms reported corresponded to those cited in UK Referral Guidelines for Suspected Cancer [28] a number of these were rarely mentioned by patients while others were frequently reported, but absent from the Guidelines. Interestingly, only five lymphoma patients reported pain after drinking alcohol.

### Strengths and weaknesses

As far as we can identify, this is the largest and most comprehensive study asking patients across all sub-types of haematological cancers (nineteen distinct diagnostic categories in total) to report their symptoms and help-seeking experiences before diagnosis. In order to limit recall bias, data were collected soon after diagnosis, with questionnaires generally being dispatched within 6 weeks of diagnosis. HMRN was established in order to facilitate research with patients and as our study was predicated on this infrastructure, we had access to a large population-based cohort of patients that had already consented to being approached for research purposes. HMRN includes patients of all ages and with all haematological malignancies, classified according to WHO schema [20], meaning that we were able to examine and compare symptoms and time-to-diagnosis across all disease sub-types, some of which had not previously been examined. Patients are asked to consent to being approached for future HMRN research with permission from their clinical team; unfortunately, some patients are deemed too ill to be approached, and others die soon after diagnosis. Consequently, the experiences of patients with very acute/aggressive or advanced stage disease (perhaps as a consequence of longer time-to-diagnosis) may not have been included in our study.

It was our intention to capture the breadth of symptoms patients considered to be related to their diagnosis; however, there are always uncertainties inherent in using self-reported data and variation may have occurred in the completeness with which patients reported their symptoms – some recording them all and others only reporting those most troublesome or painful. Use of self-reported data may also be limited by the patient’s ability to distinguish symptoms of haematological cancer from those that are unrelated, particularly at older ages when comorbidities are likely to be more common. Nonetheless it is important to take account of the patients’ interpretation of their own experiences. We are currently building on these data, however, by examining symptoms, symptom management, visit frequency and referral pathways in primary and secondary medical records, with funding from the National Awareness and Early Diagnosis Initiative [27]. Although using data from medical records has its own disadvantages, this will provide further evidence and also enable us to explore the diagnostic interval in greater detail.

### Comparison of findings with previous literature

Few studies, with the exception of those that are qualitative, have previously collected self-reported symptom-data, and ours is the largest to do this. Using this approach meant that patients could tell us their interpretation of events leading to diagnosis, and the symptoms they considered to be related to their disease. Comparing findings about time-to-diagnosis is always difficult due to different methods of data collection (self-reported survey, medical records, medical insurance claim dates), use of different summary measures (mean or median) and variation in the time-periods calculated [38]. Existing studies vary in size, with larger North American studies using SEER/Medicare data based on claims for specific symptoms [9,10]. However, average time-to-diagnosis of lymphoma (multiple types) in existing studies was reported at between 2.5 months and around a year [1,4,5,40,41]; myeloma was between 3 and 5.5 and a half months [9,29]; chronic lymphocytic leukaemia 3 months [10]; and chronic myeloid leukaemia 5 months [11]. Findings from our study are similar to these, with the exception of chronic myeloid leukaemia, which we found to be somewhat shorter at around 3 months.

Unlike existing studies, we collected information about all symptoms, rather than pre-defining the categories which would be included. Close correlation was, however, identified between the major symptoms we reported and those in existing research studies of lymphoma [7,32,35], myeloma [8,12,34], chronic myeloid leukaemia [11,33] and chronic lymphocytic leukaemia [10], although we were able to identify a far wider range.

### Implications of the study

This study provides clear evidence that time-to-diagnosis of haematological malignancies, notably myeloma and some lymphomas, can be unacceptably prolonged. The pathway to diagnosis of these cancers is reported to be more likely to include strings of repeat GP consultations [17], which are associated with a prolonged interval before hospital referral [15]; infrequent use of the urgent referral route (suggesting that malignancy is not suspected at referral) and frequent emergency presentation prior to diagnosis [16]. Previous studies of lymphoma also indicate that patients are rarely referred directly to haematology by GPs [6].

Diagnosing these diseases is undoubtedly fraught with difficulty. Symptoms are often vague and frequently seen in primary care in patients with non-malignant illness, making it difficult to differentiate patients that need urgent hospital referral from those that do not. Lack of knowledge about the symptoms of lymphoma among patients, as well as the particular characteristics of these symptoms (e.g. potentially painless, intermittent lumps) have also been reported as factors acting as barriers to help-seeking [7]. Importantly, unlike many other cancers (e.g. breast, testicular, prostate, melanoma) the symptom signature for these diseases is relatively poor; there is no single, specific symptom to prompt early help-seeking and referral. Although certain sub-types can be identified by means of a routine blood test, a specific screening test does not yet exist. In terms of UK Referral Guidelines, we have shown that these are not as useful in the context of haematological malignancy as they may be for other cancers. These factors are combined with a lack of knowledge about the impact of delayed diagnosis on outcome in these diseases, although it is recognised that patients presenting as emergencies have poorer survival than those presenting via other routes [16].

Despite the difficulties described above, however, it is important that haematological cancers are diagnosed as soon as possible, in order both to improve the patient experience and avoid increasing complications at diagnosis (such as anaemia, bone disease and renal failure in myeloma) [8]. Recent initiatives such as the UKs 'Be Clear on Cancer’ campaign have been introduced to increase knowledge of the symptoms of certain cancers among the general public. Similar approaches could be effective in the context of haematological cancers, describing for example some of the more disease specific symptoms such as the characteristics of lymphadenopathy and drenching sweats in lymphoma and bleeding and bruising in leukaemia. Further refinement of the UK Referral Guidelines for Suspected Cancer (at the very least distinguishing between myeloma, lymphoma, and the acute and chronic leukaemias) could assist GPs to identify these diseases earlier and make more timely referrals. The introduction of decision aid tools, combining information on symptoms with that of visit frequency, particularly with the same or related symptoms, may also facilitate GP decision-making.

Unfortunately, haematological malignancies are often overlooked in the context of introducing measures to promote early diagnosis. However, the findings presented in this report provide evidence that time-to-diagnosis can be unacceptably prolonged in these diseases and interventions are urgently needed to address this issue.

## Conclusion

Pathways to diagnosis are varied and can be unacceptably prolonged, particularly for myeloma and some of the lymphomas. More evidence is needed, along with interventions to reduce time-to-diagnosis, such as public education campaigns and GP decision-making aids, as well as refinement of existing Referral Guidelines.

### Ethics statement

The Haematological Malignancy Research Network has ethical approval (REC 04/01205/69) from Leeds West Research Ethics Committee, R&D approval from each Trust in the Yorkshire and Humber and Yorkshire Coast Cancer Networks, and exemption from Section 251 (formally Section 60) of the Health & Social Care Act (2001) (PIAG 1-05(h)/2007).

## Competing interests

All authors declare that they have no competing interests.

## Authors’ contributions

DH, AS, ER, RP and AJ planned the study. EM conducted the pilot study and the initial literature review. DH, ER and AS implemented the study, coded symptoms and wrote the first draft of the manuscript. AS managed and analysed the data and produced the figures. RP, AJ and UM commented on the clinical aspects of the study. UM advised on issues relating to help-seeking and diagnostic delay in the primary care setting. All authors commented on the manuscript. All authors read and approved the final manuscript.

## Pre-publication history

The pre-publication history for this paper can be accessed here:

http://www.biomedcentral.com/2052-1839/13/9/prepub
